# Disposable Voltammetric Immunosensors Integrated with Microfluidic Platforms for Biomedical, Agricultural and Food Analyses: A Review

**DOI:** 10.3390/s18124124

**Published:** 2018-11-24

**Authors:** Fabiana S. Felix, Alexandre L. B. Baccaro, Lúcio Angnes

**Affiliations:** 1 Departamento de Química, Universidade Federal de Lavras (UFLA), CP 3037, Lavras, CEP 37200-000 MG, Brazil; 2 Departamento de Química Fundamental, Instituto de Química, Universidade de São Paulo, Av. Prof. Lineu Prestes, 748, 05508-000 São Paulo, SP, Brazil; alexandre.baccaro@gmail.com (A.L.B.B.); luangnes@iq.usp.br (L.A.)

**Keywords:** voltammetric immunosensor, microfluidic devices, screen-printed electrodes, review

## Abstract

Disposable immunosensors are analytical devices used for the quantification of a broad variety of analytes in different areas such as clinical, environmental, agricultural and food quality management. They detect the analytes by means of the strong interactions between antibodies and antigens, which provide concentration-dependent signals. For the herein highlighted voltammetric immunosensors, the analytical measurements are due to changes in the electrical signals on the surface of the transducers. The possibility of using disposable and miniaturized immunoassays is a very interesting alternative for voltammetric analyses, mainly, when associated with screen-printing technologies (screen-printed electrodes, SPEs), and microfluidic platforms. The aim of this paper is to discuss a carefully selected literature about different examples of SPEs-based immunosensors associated with microfluidic technologies for diseases, food, agricultural and environmental analysis. Technological aspects of the development of the voltammetric immunoassays such as the signal amplification, construction of paper-based microfluidic platforms and the utilization of microfluidic devices for point-of-care testing will be presented as well.

## 1. Introduction

Currently, the chemical analysis of samples for clinical, environmental, food quality control, or disease-biomarkers detection is not exclusively performed in laboratories. Determinations of certain analytes contained in samples of biological fluids, e.g., blood and urine, can be performed by the patients themselves at home, using the so-called “point-of-care” diagnostic devices. 

Since the first biosensor to detect glucose in serum samples, developed by Clark and Lyons in 1962 [1], this field of research has been widely explored by different groups. To produce robust systems that integrate all the desired analytical characteristics, such as a high sensitivity and selectivity, a wide linear-operation range, low-costs of operation and assembling, capability for miniaturization to achieve the required portability, and a fast, precise and accurate detection, many repetitive studies are necessary for the several applications intended [2].

Among the different types of biosensors, the immunosensors stand out for their high specificity ascribed to the strong binding-affinity between the antibodies immobilized on the surface of the transducer and their antigens [3]. An immunosensor is considered to be the integration between the biological element and the transducer. The mutual biological interaction generates an electrical signal that is proportional to the concentration of the analyte [4].

The choice of the material of the transducer and the most appropriate proceeding to modify its surface will determine the main characteristics of the sensor, e.g., its sensitivity, selectivity and stability. The screen-printing of electrodes (SPEs) has been widely used for the construction of low-cost disposable electrochemical sensors with good reproducibility, stability and versatility of design, all of that liable to the mass production. In this sense, SPEs have emerged as an interesting alternative to assemble electrochemical immunosensors. Moreover, SPEs-based electrochemical immunosensors are suitable to the point-of-care testing, since they do not require complex instrumentation and laborious manufacturing procedures. Besides, they are inexpensive and easy to miniaturize once compared to optical and piezoelectric transducers [5]. The incorporation of nanomaterials (e.g., magnetic nanoparticles or beads, gold-nanoparticles, silica-nanoparticles, graphene, carbon-nanotubes, nanowire, quantum dots) has proved to considerably improve the electron-transfer rate, leading to an increase of the analytical signal due to the high conductivity, electrocatalytic-effect and surface area. Furthermore, an excellent biocompatibility with biological molecules has been verified [6,7]. 

The performance of the most popular electrochemical immunosensors, mainly concerning the voltammetric-type, can be further improved by the use of microfluidic platforms. They effectively consume much lower volumes of sample and chemicals during shorter analytical running-times. The integration of multiple analytical steps, like the sample preparation, the separation of analytes and their quantification, can occur under an automated circumstance inside the same device with efficiency and accuracy [8,9,10]. The potential of immunosensors is emphasized in this review, focusing on voltammetric immunoassays assembled by screen-printing strategies (SPEs) with rapid and sensitive quantification procedures in miniaturized and automated systems. Examples of applications are provided for biomedical, agricultural and food analysis. 

## 2. Voltammetric Immunosensors

Immunosensors are compact analytical devices comprising the binding between antigens and antibodies immobilized on the surface of a transducer (solid support). Antibody (Ab) is a glycoprotein that has a “Y” shaped structure with two antigen-binding sites on their upper tips. Antigen (Ag) can be a bacterium, toxin, virus, protein or any biological agent of high molecular weight (1.5 kDa). The recognition of the site of an Ag by a specific Ab forms a stable complex called epitope. An Ag contains various epitopes, but an Ab might only be bind to a specific epitope, which is the key to the high specificity of subsequent methods regarding to immunosensors [5,11]. Recently, synthetic molecules known as aptamers have been used as an alternative to Ab, since they can bind to specific biomolecules as well. However, aptamers have shown less prominent results than Ab, considering some complexities related to their structural design to attend specifically a unique Ag bonding [11,12,13].

**Figure 1:**
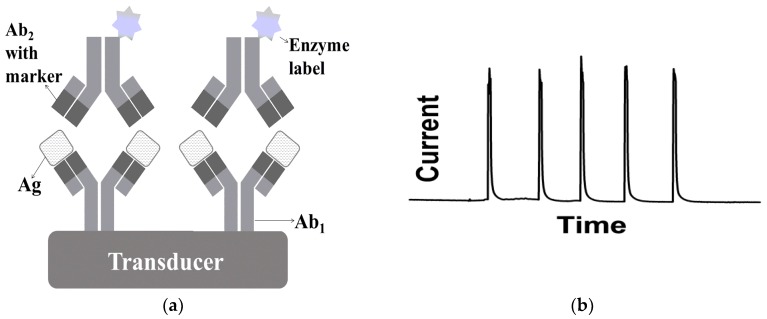
(**a**) Schematic representation of a typical electrochemical immunosensor with sandwich format and (**b**) the replicate of analytical signals obtained from injections of 10 pg·mL^−1^ ovalbumin solution during the execution of a chronoamperogram using a disposable immunosensor.

Immunosensors must show the capacity to efficiently “capture” the Ag and convert this information into an analytical signal. There are different schemes of immunoassays and the most popular is the sandwich-binding method [14]. Figure 1 shows (a) a schematic representation of a typical voltammetric immunosensor, which a specific Ag is sandwiched by two Ab (the primary and secondary Abs), and (b) the resulting analytical signals obtained with the proposed voltammetric immunosensor. 

In the sandwich-type immunosensor depicted in Figure 1, the Ag binds to two Ab: Ab_1_ is immobilized on the surface of the transducer (solid support) and Ab_2_ is labeled as an enzyme or a redox species. The enzyme or the redox species reacts with the substrate, leading to the product responsible for an indirect analytical response. Therefore, the amount of Ag is proportional to the quantity of enzyme or redox species, which reflects on the amplitude of the analytical signal obtained [15]. Compared to the competitive assay, which immobilized Ab reacts with the free Ag in competition with labeled Ag or immobilized Ag compete with free Ag for labeled free Ab, this sandwich arrangement is more sensitive (concentrations of analyte in the pmol·L^−1^ range) due to the synergistic use of the two antibodies. Nevertheless, the time of incubation is longer and it cannot be used for analytes of low molecular weight [8].

**Figure 2:**
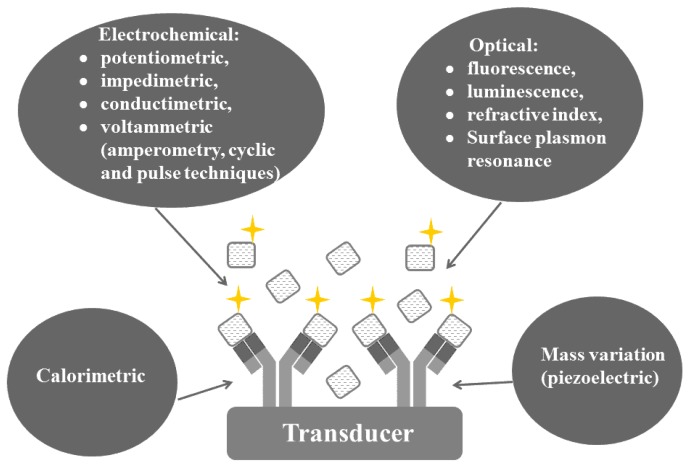
Schematic showing the classification of immunosensors according to their detection system.

Figure 2 shows different types of immunosensors that can be classified as electrochemical, optical, mass variation (piezoelectric) or calorimetric, according to their principle of detection. The electrochemical immunosensors have recently gained prominence regarding to their general characteristics, such as robustness, fast time of response, low requirement of volumes of sample, compactness, easiness and low-cost for the scale up to achieve the mass-production (modification under several different agents). They also allow the miniaturization of the analytical system, improving the portability of the device. The miniaturization of optical, calorimetric and piezoelectric immunosensors is a more challenging task for sure. They require intense light sources and monochromators apparatus (except for those operating with LEDs) and are not suited to colored and/or turbid samples [16,17]. 

Alternatively, electrochemical immunosensors allows simultaneous determination of different analytes, e.g., using multi-electrode arrays, with versatility, simplicity, quickness and under a wide range of concentrations [18,19]. Definitively, voltammetric immunosensors are among the most promising and interesting biosensors. As a rule, the biological recognition is measured by means of an electrochemical signal and, current/potential are the most commonly chosen parameters [20,21,22,23,24]. For further details, recently-published reviews might be found elsewhere [6]. The transducer of voltammetric immunosensors, also known as the working electrode, has an important role in the detection, since, additionally to providing a solid support for the immobilization of Ab or Ag, it also act as the sensing mean to detect the generated electrons from the biological interaction [25]. Therefore, the characteristics of the substrate constituting the working electrode are crucial; in particular, the analytical sensitivity is highly affected by that. Recently, screen-printed electrodes (SPE) have been widely used as the transducer for immunosensors. The screen-printing technology allows the mass production of less expensive and mechanically robust reproducible transducers [26]. 

## 3. Screen-Printed Electrodes (SPEs) for Voltammetric Immunosensors

SPEs are disposable devices assembled on chemically-inert solid substrates, using screen-printing procedures. Different configurations of SPE have been described: from a single imprinted electrode, followed by the commercial three electrodes configuration (working, reference and auxiliary electrodes) printed on the surface of the substrate that might be easily modified, (e.g., with commercial carbon or silver conductive ink) and, finally, advancing to multi-electrode arrays. Insulating materials are usually used to define the geometric area of the electrode [27,28]. 

**Figure 3:**
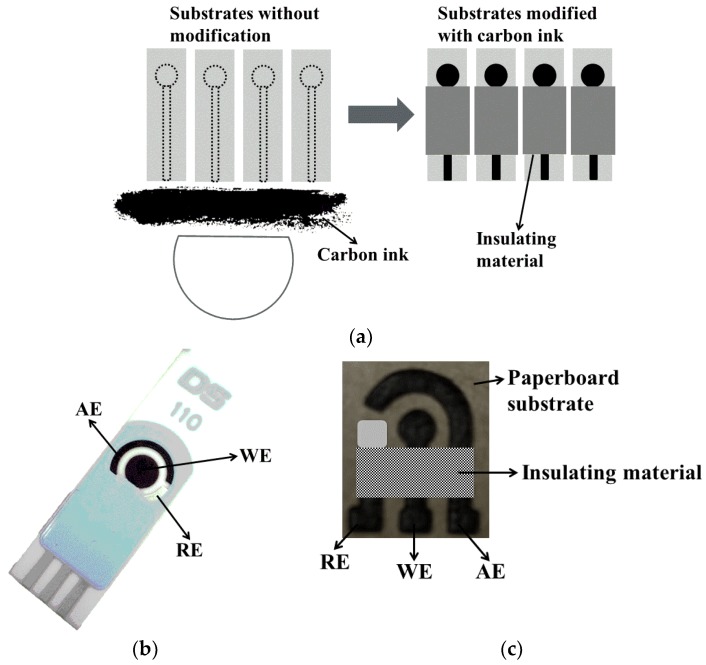
(**a**) Illustration of the process of manufacturing the SPEs using carbon ink; (**b**) commercial SPE; (**c**) paper-based analytical device (PAD) [29].

Figure 3 shows examples of SPEs: (a) schematic representation of the manufacturing process of SPEs as the working electrode; (b) a commercial SPE with three electrodes (working, auxiliary and silver pseudo reference electrodes) on a single substrate; (c) a new electrochemical paper-based analytical device (LS-ePAD) fabricated on a paperboard surface using a CO_2_ laser-scribing machine. In this last case, carbon-based material is rapidly, safely and reproducibly produced by laser-induced local pyrolysis, with or without the need of chemical reagents (only a single layer of silver ink to constitute the Ag pseudo-reference electrode [29]). 

SPEs have many advantages as transducers for immunosensors, such as: the versatility of design, good reproducibility, simple and fast process of fabrication, portability and rapid response. The miniaturization of devices considerably diminished the wasting of sample to an extent that, in some cases, it might require only a few microliters to perform the analysis. As they are disposable, these electrodes avoid some common conventional drawbacks of solid electrodes, like the memory effect and some tedious cleaning processes [26].

There are several papers reporting different applications of the SPEs as immunosensing platforms [30,31,32,33,34]. However, SPEs cannot withstand exposure to some organic solvents, which might dissolve the layer of ink and, as a consequence, a loss of sensitivity is observed [28]. 

The surface of SPEs can be easily modified with different materials (e.g., graphene, carbon nanotubes, nanoparticles, surfactants, polymers, liquid crystals and quantum dots) to improve their electrochemical performance, mainly, by increasing the surface area and improving the electron-transfer step. That results in significant magnification of the analytical signal. Particularly, graphene and carbon nanotubes have been widely used to modify immunosensors, because they efficiently confer to the working electrode these aforementioned beneficial characteristics. Eissa et al. [35] constructed voltammetric immunosensor for the detection of β-lactoglobulin in food samples (cake, sweet biscuit and cheese snacks). The surface of screen-printed carbon electrodes (SPCEs) was modified with graphene, which was electrochemically reduced to generate a monolayer of nitrophenyl groups without complete passivation of the surface. Using differential pulse voltammetry, the detection limit (LD) attained was 0.85 pg·mL^−1^.

Materials in the nanoparticle state, such as gold, silver, silica and platinum, are able to enhance the sensitivity of the immunosensors. Besides the increase of conductivity, these nanosized material offer many additional adsorption sites, increasing the interaction between the biomolecules and the surface of the transducer [36]. Lien et al. [37] proposed an electrochemical immunosensor based on the modification of SPCEs with gold nanoparticles/protein G for determination of amyloid beta, a biomarker of Alzheimer’s disease. They verified a decrease of the detection limit of, approximately, three orders of magnitude in comparison to non-modified immunosensors (from 2.04 µmol·L^−1^ to 2.65 nmol·L^−1^).

The modification of SPEs with polymers for stable and irreversible immobilization of biomolecules has been an important aspect in the development of electrochemical immunosensors. Chitosan, a linear polysaccharide, is one of the most interesting biopolymer that has been widely used in the immobilization of Ab or Ag due to its excellent film-forming ability, biocompatibility, susceptibility to chemical modification, non-toxicity, low-cost and abundance [38,39]. Brondani et al. [40] described the use of this biopolymer to immobilize the anti-cardiac troponin T antibody and stabilize the gold nanoparticles during the construction of voltammetric immunosensors. These biosensors were used for the determination of cardiac troponin T in samples of blood serum.

Silva et al. [41] incorporated thiophene monomers into the carbon ink to form a composite able to increase the sensitivity of the SPEs for the determination of dengue virus NS1 protein. Thiophene-SPE was coated by nanoparticles/protein A/anti-NS1 antibody for construction of the electrochemical immunosensor. This biosensor showed a linear range from 0.04 µg·mL^−1^ to 0.6 µg·mL^−1^ of NS1. Herein, the authors mention that thiophene is able to increase the conductivity and improve the electrochemical stability of the sensor.

**Figure 4:**
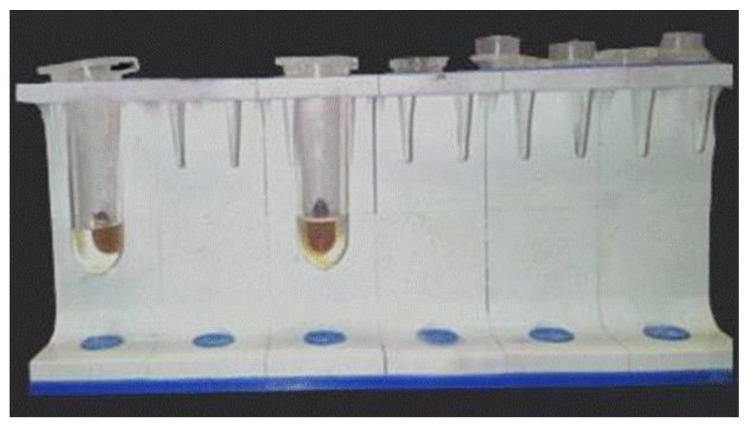
Magnetic stand used for modification of MBs during construction of an electrochemical immunosensor.

The planar surface of the SPEs facilitates its modification, as well as its coupling with the magnetic beads (MBs) by a localized magnetic field. MBs can help in lowering the detection limit, since they comprise a large surface area to immobilize biomolecules. They allow a fast and efficient purification and pre-concentration of the analyte contained in crude samples, eliminating the pretreatment or clean up steps [19,42]. In this sense, MBs have been widely used for the construction of immunosensors to improve the sensitivity and selectivity of the analytical methods, and minimize interferences from the matrix of complex samples. Figure 4 shows a magnetic stand used for modification of MBs with antibodies/horseradish peroxidase during the construction of an electrochemical immunosensor for ovalbumin analyses. 

**Figure 5:**
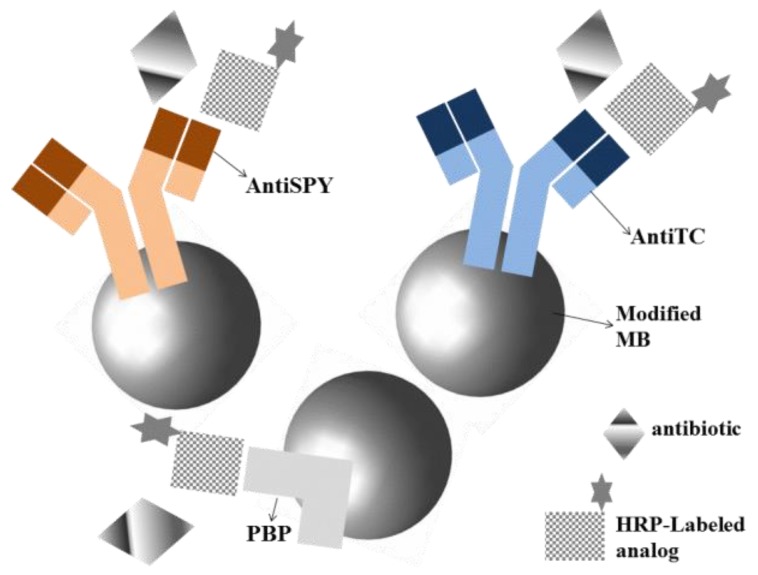
Schematic representation of the competitive assay with MBs modified with AntiTC, AntiSPY and PBP for construction of voltammetric immunosensor. AntiTC = antibodies for tetracyclines, AntiSPY = antibodies for sulfonamides and PBP = His_6_-tagged penicillin-binding protein for cephalosporins [23].

Conzuelo et al. [23] described for the first time the use of voltammetric immunosensors for sensitive multiplexed detection of residues of cephalosporins, sulfonamides and tetracyclines antibiotics in milk matrices. Using MBs modified with a mixture of specific targets (antiTC = antibodies for tetracyclines, antiSPY = antibodies for sulfonamides and PBP = His_6_-tagged penicillin-binding protein for cephalosporins) and direct competitive assays with SPE as the transducer, it was possible to record the extent of different affinity reactions at −0.20 V (vs. Ag pseudo-reference electrode) in presence of H_2_O_2_ and hydroquinone (Figure 5). 

Disposable immunosensor arrays for simultaneous electrochemical analysis are also currently being explored with sandwich assays. Recently, Munge et al. [18] have reviewed the use of multiplex immunosensor arrays for quantification of cancer biomarker proteins. The authors discuss different strategies, including the enzyme-based immunoarrays, nanoparticle-based immunoarrays, electrochemical and electrochemiluminescence methods, to detect protein biomarkers for clinical diagnosis in a faster and more sensitive way, for both competitive and sandwich assays. Alonso-Lomillo and Dominguez-Renedo have also recently highlighted in a review the different strategies of immobilization of biomolecules on SPEs for drug analysis [43].

The association of SPE with flow systems (mainly flow injection analysis—FIA) can still increase the reproducibility, sensitivity and reduce the time of analysis of electrochemical immunosensors. FIA is a well-established technique, which a plug of the sample containing the analyte is injected into a continuously-flowing carrier solution that, depending on the main characteristics of the system conferred by the manifold profile, might dilute or concentrate the analyte before reaching a detector with great precision, accuracy and speed [44]. 

Okadaic acid (OA) is a toxin produced by various species of dinoflagellates and can cause food poisoning in humans after consumption of contaminated molluscs [45]. An automated flow-through electrochemical immunosensor was developed for OA determination in mussel samples. During amperometric experiments, MBs modified with OA were injected onto SPCE in the flow system. From an indirect competitive immunoassay, it was possible to obtain a linear range of 0.19 to 25 µg·L^−1^ for okadaic acid [46].

Wu et al. [47] described a disposable voltammetric immunosensor associated with FIA for detection of carcinoembryonic antigen (CEA), an important tumor marker. SPCE modified with CEA/colloidal Au/chitosan film was used for the assembling of a competitive immunoassay, which was used in association with the flow injection analysis. Linear responses were obtained in the range of 0.50 to 25 ng·mL^−1^ (LD = 0.22 ng·mL^−1^) using a flow rate of 3.6 mL·min^−1^.

**Figure 6:**
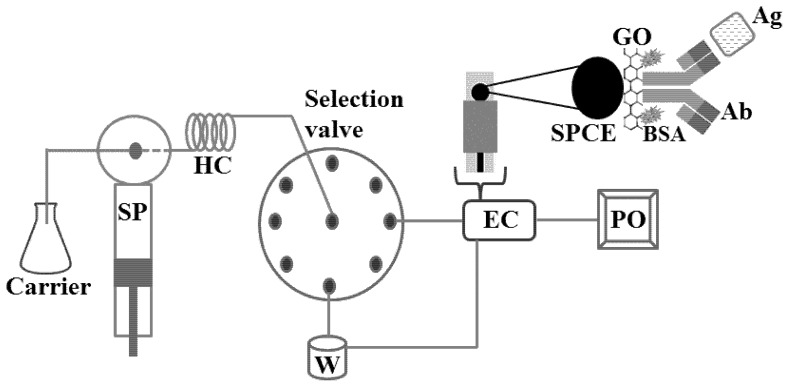
Basic scheme of SIA manifold used for quantification of HIgG with electrochemical immunosensor constructed on SPCE modified with graphene. SP = syringe pump; HC = holding coil; W = waste; EC = electrochemical cell containing the immunosensor; PC = personal computer; GO = graphene oxide; BSA = bovine serum albumin; PO = potentiostat [48].

Thunkhamrak et al. [48] developed a voltammetric immunosensor for quantification of human immunoglobulin G (HIgG), an important biomarker of many diseases such as Alzheimer’s disease and cancers. The authors used the SPCE modified with graphene oxide for immobilization of anti-HIgG and, afterwards, this sensor was placed in a flow cell for the sequential injection analysis (SIA), with a flow rate of 2 mL·min^−1^ (Figure 6). SIA is based on similar principles of FIA, despite of the possibility of bidirectional flow sense: precise injection of sample, controlled dispersion of the sample zone and high sampling frequency. SIA uses a multi-position valve automatically controlled by a computer [44,49], which provides a great versatility to the operator at the cost of partially sacrificing the analytical frequency. Table 1 shows some examples of voltammetric immunosensors fabricated on SPE and associated with flow systems (FIA or SIA).

## 4. Association of Microfluidic Devices and Disposable Voltammetric Immunosensors

During the planning of immunosensors experiments and their design, it should be accounted the integration of the transducer with a microfluidic system. This combination arises many advantages, such as the reduction of the elapsed time of experiment, the sample, reagent and power consumption, and the risk of contamination. On the other hand, it favors the portability, increasing the sensibility and reliability of resulting data, all of that due to the automation and integration of multiple processes on a single device [8,18]. Moreover, biomolecules, such as Ag and Ab have been considered as promising elements, which can be integrated with microfluidic platforms to conduct molecule-specific capture and to promote the complete separation of targeted analytes, in a fast and secure way.

The idea of microfluidic analytical devices arose from the concept of Total Analysis System (TAS), referred also as “lab-on-a-chip” or “micro-total-analysis-system” (µTAS), which includes a whole setup of a laboratory onto a single chip, or channels in microscale. These devices are mainly composed of actuators (micropumps, microvalves or micromixers) and sensors for any type of detection system, including electrochemical, optical, among others [54]. The microfluidic systems generally are constructed using techniques from high tech industries, which comprises the association between photolithography, silicon microelectronics and microelectromechanical systems, employing different materials (glass, silicon and/or polymers). The thermoplastic polymers are the most common materials implemented [55,56]. In addition to choosing the best materials and designing the microchannels, additional aspects must be taken into account: the introduction of samples into the device, the propulsion of solution inside the channels, the effects of modification on the microchannel walls, the effect of the immobilization of biomolecules onto microfluidic platform (or on the transducer surface) and, finally, the optimization of the detection system [57]. Many works have been reported with microfluidic systems to describe their advantages in different approaches, such as the interaction studies between cells, the different forms of miniaturization of enzymatic reactors and the signal amplification strategies for immunoassays in different detection systems [58,59,60,61].

In 2009, a new analytical approach involving ELISA and microfluidic platform for quantification of a mycotoxin from Fusarium species in food samples was reported [62]. In this study, with an amperometric detector, it was possible to observe a detection limit of 0.83 µg·L^−1^. Two years later, this analytical system was fully integrated into a microfluidic chip. The same authors obtained a detection limit of 0.40 µg·L^−1^, half of the previously reported value, by using a competitive immunoassay [63].

**Figure 7:**
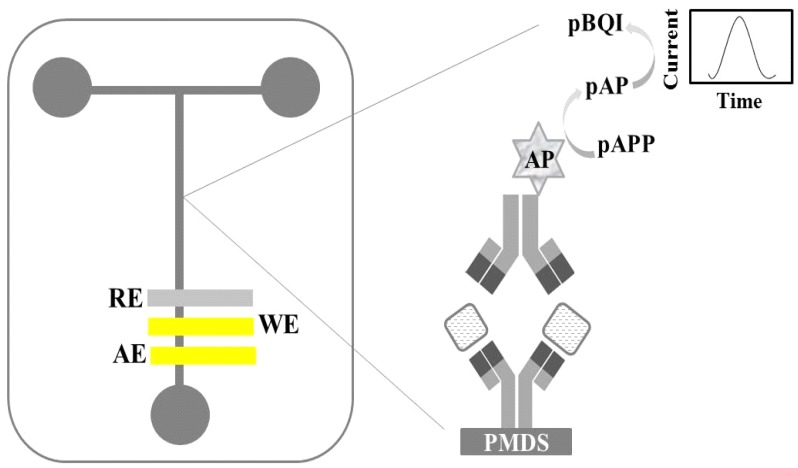
Schematic representation of microfluidic voltammetric immunosensor constructed for determination of mycotoxin in walnut samples. WE = working electrode, RE = reference electrode, AE = auxiliary electrode, PMDS = poly(dimethylsiloxane) modified with glass particles, AP = alkaline phosphatase enzyme, pAPP = *p*-aminophenyl phosphate, pAP = *p*-aminophenol, pBQI = *p*-benzoquinone imine [64].

Regiart et al. [64] reported a microfluidic amperometric immunosensor for quantification of Xanthomonas arboricola mycotoxin (XA) in walnut samples. The authors constructed a sandwich immunoassay in the central channel of glass-poly (dimethylsiloxane) material (Figure 7). XA was quantified by the amperometric measurements based on the conversion of *p*-aminophenyl phosphate (p-APP) to *p*-aminophenol (p-AP), in the presence of alkaline phosphatase enzyme (AP), then the enzymatic product was oxidized to *p*-benzoquinone imine (p-BQI) at 100 mV on a gold electrode prepared by the sputtering method.

Microfluidic voltammetric immunosensors can be constructed in homogeneous or heterogeneous assays. The second ones are the most commonly applied for applications. The main differences between these two configurations, their applications, advantages, disadvantages as well as the differences in the protocols of immobilization of the biomolecules on the solid substrate are highlighted and discussed in details by Hervas et al. [65]. 

Voltammetric immunosensors possess a great potential for integration with microfluidic systems, considering their low detection limits, the wide variety of analytes that might be detected (e.g., drugs, pesticides, protein biomarkers and mycotoxins) and the ease of implementation with low-costs. In addition, the analytical responses of these biosensors are due to reactions over the interface of the transductor, making the detection system much more independent of the electrochemical cell volume than optical immunosensors (optical length). Voltammetric analysis can be performed with simple, portable and non-expensive equipment: a potentiostat, and, even by that, it still comprises high sensitivity. Nonetheless, one of the major challenges for these immunosensors is the sample preparation step, particularly for biological matrices such as blood, since some of its components interfere with the analytical response, and consequently, impair the immunoassay performance.

Microfluidic paper-based analytical devices (µPADs) or “paper-based microfluidics” have attracted a lot of attention due to their inherent characteristics such as the ease of use, low-cost, reproducibility, repeatability and disposability. These characteristics might still be aligned with the advantages of the microfluidic technologies of short time of analysis, low consumption of sample and chemicals, reliability of results, increased sensitivity and portability of devices. Moreover, paper is a cheap, biodegradable and naturally abundant material, and it is simple to be chemically modified [66,67,68].

In 2007, Whiteside’s group reported a microfluidic paper-based analytical device for bioassays. In their first paper, the authors demonstrated the possibility of the simultaneous detection of glucose and protein in urine. The proposed platform is simple, disposable, inexpensive and portable and it can be considered a feasible alternative for bioanalysis, mainly, in emerging countries [69]. In 2009, Dungchai et al. [70] published, for the first time, a voltammetric paper-based microfluidic device for quantification of glucose, lactate and uric acid in biological samples. In this study, screen-printed electrodes were closely positioned to the paper and modified with Prussian blue to improve their selectivity.

The sampling of biological fluids and the detection of analytes are steps usually carried out totally apart one each other. Small and reliable microfluidic devices for point-of-care testing (POCT, analytical procedures performed in the presence of or by the patient itself) can be a more accessible alternative for clinical diagnostic. These methods are user-friendly and require small volumes of samples that are analyzed in shorter times, avoiding errors generated by the manipulation and stocking [71]. The coupling of POCT and µPADs produced plenty of advantages in many applications, since the capillary force is explored for the propulsion of the sample within the device instead of conventional pumps [72]. 

Recently, Wang et al. [73] have reported the use of microfluidic devices for the voltammetric quantification of the hormone 17-estradiol. For these experiments, the microfluidic channel was fabricated with wax printing and a SPE platform provided the three electrodes for the voltammetric process. To improve the performance of the working electrode, it was modified with carbon nanotubes and gold nanoparticles to accelerate the electron-transfer rate. This modification provided a linear range from 0.01 to 100 ng·mL^−1^. The authors suggested the use of this integrated microfluidic-biosensor platform for a POCT.

In their review, Barbosa and Reis [74] have pointed out the poor adsorption of microfluidic devices in association with POCT for the analysis of protein biomarker. Authors covered the period from 2005 to 2016 and claim that these systems were still inefficient in comparison with conventional sophisticated pathology tests. One of the main reasons for that is the lack of portable microfluidic devices with low-cost techniques. Nowadays, a better understanding of the immunoassays associated with improved manufacturing processes of portable microfluidic tools are required to stimulate the commercial disposable integrated immunosensors-microfluidic platform market. Better strategies for immobilization of antibodies or antigens on the substrate, methods for enhancing antigen-antibody interaction, sample preparation, detection modes and signal amplification systems are currently under studies to allow these analytical devices to be more widely used in the early diagnosis of chronic diseases, such as hypertension, cancer and hypercholesterolemia. Other non-clinical possible applications include the detection of residues of pesticides in water, soil and food, and the detection of mycotoxins in food.

In general, the combination of microfluidic platforms with disposable voltammetric immunosensors has still been underexplored for construction of sensing devices. The most popular detection mode is the fluorescence. A brief summary of this subject is given in Table 2 for biomedical, food, agricultural food and environmental applications. 

Few papers describe the development of microfluidic devices that use a non-voltammetric technique of detection. An impedimetric immunosensor based on a microfluidic chip has been used for the determination of chlorpyrifos, an organophosphorus pesticide, in vegetable samples [96]. In this study, an array of gold microelectrodes was modified with PDDA/AuNPs/Protein A for the immobilization of the anti-chlorpyrifos monoclonal antibody. A linear range was obtained from 0.5 ng·mL^−1^ to 500 ng·mL^−1^. Tang et al. [97] used microfluidic chips for the construction of potentiometric immunosensors. The transducers were modified with NiFeO_4_/SiO_2_ nanoparticles before immobilization of the antibodies for simultaneous quantification of four tumor markers (AFP, CEA, CA 125 and CA 15-3). These magnetic nanoparticles altogether with a local magnetic field selectively retained the analytes.

## 5. Conclusions

Voltammetric immunosensors in association with microfluidic platforms are attracting great interest due to their great potential for analytical applications. Aspects such as their elevated sensitivity, exceptional selectivity, rapid response, good reproducibility, simple and fast assembling, possibility of miniaturization, low consumption of chemicals and samples, and portability are some of their advantages. This review shows that microfluidic voltammetric biosensors constructed using screen-printing technology are interesting analytical tools for fast, selective and sensitive quantification of different analytes, including cancer biomarkers, antibiotics, pesticides, mycotoxins and hormones. The incorporation of nanomaterials such as graphene, carbon-nanotubes and metallic nanoparticles has led to an improvement in sensitivity and reproducibility of these immunosensors. The integration of voltammetric paper-based analytical devices and the execution of immunoassays in microfluidic systems can create versatile platforms for construction of reliable, disposable, low-cost and portable devices for point-of-care testing. These disposable systems have demonstrated the potentiality to detect low concentration of analytes (down to fg·mL^−1^) in complex samples using miniaturized and reliable set-ups. Even with continuous growth of the voltammetric immunosensors on microfluidic platforms, they still remain incipient for commercialization. From our point of view, by the efforts of researchers in the field, these devices will receive great importance in the near-to-medium future, especially for point of care testing devices. We also believe that the simultaneous detection of different analytes with a single integrated microfluidic-immunosensor device will be a common practice. 

## Figures and Tables

**Table 1:** Voltammetric immunosensors constructed on SPE and associated with flow analysis in different applications.

Analyte	Transducer	Detection Limit	Sample	Reference
Okadaic acid (toxin)	SPCE modified with MBs	0.15 µg·mL^−1^	Mussel	[46]
CEA (tumor marker)	SPCE modified with CEA/colloidal Au/chitosan film	0.22 ng·mL^−1^ 0.45 ng·mL^−1^	Serum	[47]
HIgG (biomarker)	SPCE modified with GO	1.70 ng·mL^−1^	Urine	[48]
Isoproturon (herbicide)	SPCE	0.84 ng·mL^−1^	Soil	[50]
2,4-D (herbicide)	Gold-SPE modified with cysteamine	0.12 µg·mL^−1^	Water and food	[51]
Biotin (vitamin)	SPCE	10^−14^ mol·L^−1^	Clinical	[52]
*Botrytis cinerea* (fungus)	SPCE modified with MWCNT	0.02 µg·mL^−1^	Fruit	[53]

**Abbreviations:** CEA = carcinoembryonic antigen; HIgG = human immunoglobulin G; GO = graphene oxide; 2,4-D = 2,4-dichlorophenoxyacetic acid; MWCNT = multi-walled carbon nanotubes.

**Table 2:** Examples of applications of microfluidic devices in the construction of voltammetric immunosensors on SPEs.

Application	Analyte	SPE Modified with	LOD	Reference
Biomedical	17-E2 (hormone)	MWCNTs/THi/AuNPs	10 pg·mL^−1^	[73]
	CA15-3 (biomarker)	-	6.0 µU·mL^−1^	[75]
	ER α (biomarker)	PDDA/GSH-AuNPs/DNA	10 fg·mL^−1^	[76]
	PSA (biomarker)	Glutathione/AuNPs	0.23 pg·mL^−1^	[77]
	IL-6 and IL-8 proteins (biomarkers)	AuNPs	5.0 fg·mL^−1^ (IL-6) and 7.0 fg·mL^−1^ (IL-8)	[78]
	NSE (biomarker)	NH_2_-G/THi/AuNPs	10 pg·mL^−1^	[79]
	HCG (biomarker)	-	0.36 mIU·mL^−1^	[80]
Biomedical	*E. coli* (bacteria)	AuNPs	50 cfu·mL^−1^ and 2.0 × 10^4^ cfu·mL^−1^ (standard immunoassay)	[81]
	CEA, CA 19-9, H.P., P53, PGI and PGII (biomarkers)	-	0.37 ng·mL^−1^ (CEA), 10.75 U·mL^−1^ (CA 19-9), 5.0 U·L^−1^ (H.P.), 35 pg·mL^−1^ (P53), 37.5 ng·mL^−1^ (PGI) and 2.5 ng·mL^−1^ (PGII)	[82]
	H_1_N_1_ (virus)	rGO/EDC-NHS	0.5 PFU·mL^−1^	[83]
	TNFα (Biobmarker)	-	4.1 ng·mL^−1^	[84]
	AFP (biomarker)	rGO-TEPA/AuNPs	0.005 ng·mL^−1^	[85]
	GMN (aspergillosis)	CuNPs-PVP	0.23 ng·mL^−1^	[86]
	Dengue NS1 protein	-	0.5 ng·mL^−1^	[87]
	Tetracycline and pristinamycin (antibiotics)	-	6.33 ng·mL^−1^ (tetracycline) and 9.22 ng·mL^−1^ (pristinamycin)	[88]
	CA 125, CEA (biomarkers)	MWCNTs	0.2 mU·mL^−1^ (CA 125) and 0.01 ng·mL^−1^ (CEA)	[89]
	PfHRP2 (biomarker)	-	16 ng·mL^−1^	[90]
	BAM (herbicide)	-	Not found	[91]
Food	CLB (2-agonist)	AuNPs	0.008 ng·mL^−1^	[92]
	*S. typhi* (bacterium)	-	7.7 cells·mL^−1^	[93]
Agricultural Food	XA (toxin)	-	1.5 × 10^2^ CFU·mL^−1^	[64]
	OTA (mycotoxin)	-	0.05 µg·Kg^−1^	[94]
	*B. cinerea* (fungus)	-	0.008 µg·mL^−1^	[95]

**Abbreviations:** 17-E2 = 17-estradiol; CA15-3 = carbohydrate antigen 15-3; ERα = estrogen receptor α; PSA = prostate specific antigen; IL-6 = interleukin-6; IL-8 = interleukin-8; NSE = neuron-specific enolase; HCG = human chorionic gonadotropin; CEA = carcinoembryonic antigen; CA 19-9 = carbohydrate antigen 19-9; H.P. = *Helicobacter pylori* CagA protein; PGI = pepsinogen I; PGII = pepsinogen II; H_1_N_1_ = human influenza A; TNFα = tumor necrosis factor alpha; AFP = α-fetoprotein; GMN = galactomannan; dengue NS1 = non-structural proteins; CA 125 = carcinoma antigen 125; PfHRP2 = *Plasmodium falciparum* histidine-rich protein 2; BAM = 2,6-dichlorobenzamide; CLB = clenbuterol; S.typhi = *Salmonella typhimurium*; XA = *Xanthomas arboricola*; OTA = ochratoxin A; B. cinerea = *Botrytis cinerea*; P53 = P53 oncoprotein; CuNPs = copper nanoparticles; PVP = polyvinylpyrrolidone; GSH = reduced L-glutathione; THi = thionine; PDDA = poly(diallyldimethylammonium chloride); NH_2_-G = amino functional graphene; AuNPs = gold nanoparticles; rGO = reduced graphene oxide; TEPA = tetraethylene pentamine; EDC-NHS = N-ethyl-N-(3-dimethylaminopropyl) carbodiimide/N-hydroxysuccinimide. chloride); NH_2_-G = amino functional graphene; AuNPs = gold nanoparticles; rGO = reduced graphene oxide; TEPA = tetraethylene pentamine; EDC-NHS = N-ethyl-N-(3-dimethylaminopropyl) carbodiimide/N-hydroxysuccinimide.
